# Community Well‐Being and Functional Disability Risk Among Older Adults: A Multilevel Analysis of 91 Municipalities in Japan

**DOI:** 10.1111/ggi.70337

**Published:** 2026-01-21

**Authors:** Tomoki Tanaka, Weida Lyu, Katsuya Iijima

**Affiliations:** ^1^ Institute of Gerontology The University of Tokyo Tokyo Japan; ^2^ Institute for Future Initiatives The University of Tokyo Tokyo Japan

Dear Editors,

1

In advanced nations experiencing rapid population aging, such as Japan, maintaining supportive community environments and sustainable living conditions for older adults are urgent priorities [1]. Frailty, characterized by the accumulation of physical, psychological, and social function decline, is a reversible condition and an important target for preventing adverse health outcomes [2]. Previous practices have primarily focused on individual‐level risk factors; the role of community‐level environments in shaping trajectories remains underexplored [3]. The Community Well‐Being Index (CWBI), developed by the Digital Agency of Japan, is an objective, multidimensional measure of municipal livability [4]. However, few studies have empirically linked community‐level well‐being to frailty‐related risk among older adults at a national scale. We aimed to examine the association between community well‐being and the frailty checkup risk score (FCRS), a composite indicator of functional disability reflecting physical and social domains derived from the frailty checkup (FC) program [5].

Data were obtained from 91 municipalities participating in the FC program between 2018 and 2024. The study included 37 208 community‐dwelling adults aged ≥ 65 years (mean age 76.6 ± 7.2 years; 77% female) with complete demographic data. The outcome variable was the FCRS, which represents the degree of frailty progression encompassing physical and social domains [5]. The primary exposure of interest was community‐level well‐being, operationalized using domain‐specific scores from the 2024 CWBI, derived from objective administrative indicators [4]. Individual‐level covariates (Level 1) included age, sex, and self‐efficacy for health management, which measures confidence in performing multiple health‐related behaviors [6]. Community‐level covariates (Level 2) were incorporated as contextual predictors in the multilevel model. A generalized linear mixed model with municipalities as random intercepts was employed, specifying a normal distribution and identity link. Analyses were performed using SPSS version 29.0 (IBM Japan, Tokyo).

At the individual level, older age was associated with higher FCRS, whereas male sex and higher self‐efficacy for health management were associated with lower FCRS (*p* < 0.001 for all; Table 1). At the community level, several CWBI domains demonstrated significant associations. Municipalities with higher shopping and dining convenience, digital life and childcare environments, and environmental symbiosis showed significantly lower FCRS. Municipalities with higher public space, diversity and tolerance, employment and income, business creation, and educational environment scores showed higher FCRS. Sixteen other indicators were non‐significant. Between municipality variance was significant (variance = 0.284, *p* < 0.001), and the intraclass correlation coefficient (ICC, 0.38) indicated that approximately 38% of the total FCRS variance was attributable to intermunicipality differences or unmeasured individual‐level factors shared within municipalities.

**TABLE 1:** Multilevel analysis of individual‐ and community‐level factors associated with the frailty checkup risk score among older adults across 91 municipalities (*n* = 37 208; 36, 32, 6, 3, 5, 6, and 3 in the Kanto, Chubu, Kinki, Chugoku, Shikoku, Kyushu, and Okinawa, and Tohoku regions, respectively).

Variable	Mean (SD)	*β* (95% CI)^a^	*p*
Level 1: Individual factors			
Age, years	76.4 (7.1)	0.055 (0.054 to 0.056)	< 0.001
Sex, the percentage of male	23.0%	−0.251 (−0.273 to −0.230)	< 0.001
Self‐efficacy for health care^b^	48.1 (7.4)	−0.051 (−0.073 to −0.030)	< 0.001
Level 2: Community well‐being indicators^c^			
Community‐level indicators that are potentially protective against frailty			
Shopping and dining convenience	48.7 (3.7)	−0.028 (−0.034 to −0.021)	< 0.001
Digital life environment	46.3 (7.6)	−0.004 (−0.008 to −0.001)	0.024
Childcare environment	49.5 (3.8)	−0.004 (−0.007 to −0.001)	0.018
Environmental symbiosis	50.4 (4.0)	−0.010 (−0.013 to −0.006)	< 0.001
Local government services	50.5 (4.6)	−0.003 (−0.006 to 0.000)	0.090
Community‐level indicators associated with higher risk or inclusive urban contexts			
Transportation and mobility	48.7 (4.6)	0.011 (0.007 to 0.016)	< 0.001
Public space	47.1 (6.2)	0.013 (0.010 to 0.016)	< 0.001
Diversity and tolerance	49.3 (6.1)	0.006 (0.003 to 0.009)	< 0.001
Employment and income	50.5 (5.0)	0.007 (0.004 to 0.010)	< 0.001
Business creation	48.4 (4.6)	0.010 (0.006 to 0.014)	< 0.001
Community self‐efficacy	54.0 (16.0)	0.004 (0.002 to 0.006)	< 0.001
Primary and secondary education	50.4 (4.9)	0.006 (0.003 to 0.010)	< 0.001
Nonsignificant indicators			
Health and welfare	50.8 (3.5)	0.000 (−0.005 to 0.005)	0.982
Housing environment	52.2 (9.2)	0.001 (−0.003 to 0.005)	0.756
Leisure and recreation	50.4 (7.7)	0.001 (0.000 to 0.003)	0.122
Urban landscape	49.5 (11.6)	0.000 (−0.001 to 0.001)	0.810
Safety (accidents and crime)	52.3 (6.7)	−0.002 (−0.004 to 0.001)	0.208
Natural scenery	46.4 (8.3)	0.001 (−0.001 to 0.002)	0.462
Natural resources	51.0 (7.4)	0.000 (−0.003 to 0.003)	0.979
Natural disasters	50.8 (3.9)	−0.001 (−0.006 to 0.003)	0.615
Community connectedness	51.2 (5.8)	−0.002 (−0.008 to 0.004)	0.481
Health status (community‐level)	53.7 (7.5)	0.000 (−0.002 to 0.002)	0.747
Culture and arts	49.3 (4.1)	−0.003 (−0.007 to 0.001)	0.116
Educational opportunities	49.9 (4.0)	0.000 (−0.004 to 0.005)	0.892

Abbreviations: CI, confidence interval; SD, standard deviation.

^a^ Regression coefficients (*β*) were estimated using a generalized linear mixed model with the frailty checkup risk score (FCRS) as the dependent variable. The model specified municipalities as random intercepts and included both individual‐level (Level 1) and community‐level (Level 2) predictors. A normal distribution with identity link was applied after evaluating model fit; a variance component covariance structure was used for the random effects. Municipal random effect variance = 0.284 (*p* < 0.001); the intraclass correlation coefficient (ICC) was 0.38, indicating that approximately 38% of the total variance in FCRS was attributable to inter‐municipality differences or unmeasured individual‐level factors shared within municipalities.

^b^ Self‐efficacy for health management was assessed using a validated scale measuring confidence in performing multiple health‐related behaviors, including maintaining a balanced diet, regular exercise, stress management, social participation, medical adherence, and disease prevention.

^c^ The Community Well‐Being Index (CWBI) developed by the Digital Agency of Japan. CWBI domain scores are standardized objective administrative indicators with a national mean of 50, where higher scores indicate more favorable relative conditions. Although the CWBI was originally designed to integrate both objective indicators and survey‐based subjective measures, subjective data were unavailable or insufficiently representative in many municipalities as of 2024; therefore, only objective indicators were used in this study. The “Transportation and mobility” domain reflects accessibility and walkability of daily transportation infrastructure rather than vehicle ownership alone. Community‐level self‐efficacy represents aggregated contextual characteristics of municipalities and is conceptually distinct from individual‐level self‐efficacy.

This nationwide multilevel analysis suggested that community‐level well‐being remained significantly associated with frailty‐related risk, despite adjusting for individual characteristics. Municipalities with greater lifestyle convenience, information and communication technology accessibility, and environmentally conscious development had lower FCRS; thus, supportive environments may help maintain daily autonomy and social engagement. Conversely, higher FCRS in municipalities with greater public space, diversity, and economic vitality might reflect inclusive urban contexts—areas where older adults with diverse health conditions, including frailty, are more visible and socially integrated. These findings indicate that community well‐being is not merely a measure of livability but also a contextual factor shaping population health through both protective and inclusive mechanisms. These domains likely reflect broader social and infrastructural contexts rather than isolated facilities.

The ICC suggested that nearly 40% of the total variance in frailty risk was attributable to municipality‐level differences or unmeasured shared individual factors, such as social capital or access to services, aligning with the World Health Organization's Integrated Care for Older People framework [7], which emphasizes environmental and social structures as essential to maintaining intrinsic capacity and functional ability. Similarly, the Asian Working Group for Sarcopenia 2025 highlighted the importance of community‐level and environmental approaches to muscle health and frailty prevention across the life course [8]. These frameworks support the interpretation that age‐friendly multidimensional environments may mitigate frailty progression in super‐aged societies.

This study has some limitations. Its cross‐sectional design precludes causal inference. Participants were community‐dwelling older adults who participated voluntarily; therefore, the sample may not fully represent all residents in each municipality. Although the FCRS integrates multiple individual‐level functional and health‐related components, detailed clinical diagnoses and comorbidity data were not available, and such unmeasured health factors may have influenced the results. Moreover, community indicators are based on objective municipal averages, which may not reflect within‐community diversity or subjective perceptions of livability. Future longitudinal studies integrating subjective well‐being and neighborhood‐level data are warranted to clarify causal pathways and heterogeneity.

In conclusion, community well‐being was independently associated with frailty‐related risk among older adults. Strengthening digital, environmental, and social infrastructures may serve as a foundation for community‐based frailty prevention.

## Author Contributions

**Tomoki Tanaka:** writing – original draft, visualization, validation, supervision, software, resources, project administration, methodology, investigation, funding acquisition, formal analysis, data curation, conceptualization. **Weida Lyu:** writing – review and editing, visualization, validation, software, methodology, investigation, funding acquisition, formal analysis, data curation. **Katsuya Iijima:** writing – review and editing, visualization, validation, supervision, project administration, methodology, investigation, formal analysis, data curation, conceptualization.

## Funding

The authors have nothing to report.

## Ethics Statement

The study protocol was approved by the Ethics Committee of the University of Tokyo(#19–320), and written informed consent was obtained from all participants. The study was conducted following the principles of the Declaration of Helsinki.

## Conflicts of Interest

The authors declare no conflicts of interest.

## Data Availability

The data that support the findings of this study are available from the corresponding author upon reasonable request.
